# Evidence based on Mendelian randomization and colocalization analysis strengthens causal relationships between structural changes in specific brain regions and risk of amyotrophic lateral sclerosis

**DOI:** 10.3389/fnins.2024.1333782

**Published:** 2024-03-05

**Authors:** Jiaying Shi, Zhaokun Wang, Ming Yi, Shengyu Xie, Xinyue Zhang, Dachang Tao, Yunqiang Liu, Yuan Yang

**Affiliations:** Department of Medical Genetics, State Key Laboratory of Biotherapy, West China Hospital, Sichuan University, Chengdu, China

**Keywords:** Mendelian randomization, colocalization, brain structures, amyotrophic lateral sclerosis, causality, genome-wide association study

## Abstract

**Background:**

Amyotrophic lateral sclerosis (ALS) is a neurodegenerative disease characterized by the degeneration of motor neurons in the brain and spinal cord with a poor prognosis. Previous studies have observed cognitive decline and changes in brain morphometry in ALS patients. However, it remains unclear whether the brain structural alterations contribute to the risk of ALS. In this study, we conducted a bidirectional two-sample Mendelian randomization (MR) and colocalization analysis to investigate this causal relationship.

**Methods:**

Summary data of genome-wide association study were obtained for ALS and the brain structures, including surface area (SA), thickness and volume of subcortical structures. Inverse-variance weighted (IVW) method was used as the main estimate approach. Sensitivity analysis was conducted detect heterogeneity and pleiotropy. Colocalization analysis was performed to calculate the posterior probability of causal variation and identify the common genes.

**Results:**

In the forward MR analysis, we found positive associations between the SA in four cortical regions (lingual, parahippocampal, pericalcarine, and middle temporal) and the risk of ALS. Additionally, decreased thickness in nine cortical regions (caudal anterior cingulate, frontal pole, fusiform, inferior temporal, lateral occipital, lateral orbitofrontal, pars orbitalis, pars triangularis, and pericalcarine) was significantly associated with a higher risk of ALS. In the reverse MR analysis, genetically predicted ALS was associated with reduced thickness in the bankssts and increased thickness in the caudal middle frontal, inferior parietal, medial orbitofrontal, and superior temporal regions. Colocalization analysis revealed the presence of shared causal variants between the two traits.

**Conclusion:**

Our results suggest that altered brain morphometry in individuals with high ALS risk may be genetically mediated. The causal associations of widespread multifocal extra-motor atrophy in frontal and temporal lobes with ALS risk support the notion of a continuum between ALS and frontotemporal dementia. These findings enhance our understanding of the cortical structural patterns in ALS and shed light on potentially viable therapeutic targets.

## 1 Introduction

Amyotrophic lateral sclerosis (ALS) is a rapidly progressing neurodegenerative disease characterized by loss of motor neurons. Familial ALS accounts for approximately 10% of ALS cases, with the remaining 90% being classified as sporadic ALS (Goutman et al., 2022). Patients typically experience asymmetrical muscle weakness, dysarthria, dysphagia and respiratory failure due to the progressive loss of upper (UMN) and lower motor neurons (LMN) affecting bulbar, cervical, thoracic, and/or lumbar segments (Goutman et al., 2022). The underlying mechanisms of the disease are not well understood. Recent research suggests that a combination of genetic susceptibility and external triggers might contribute to the development of ALS (Al-Chalabi et al., 2014, 2016). Currently, the diagnosis largely relies on clinical observations of UMN and LMN signs in the same affected area, supported by neurophysiological testing and MRI findings to rule out other conditions. The varying degrees of UMN and LMN involvement result in the clinical variability, ranging from progressive muscular atrophy (exclusive LMN involvement) to primary lateral sclerosis (exclusive UMN involvement). As a result of this disease spectrum, individual prognoses vary significantly, with survival durations ranging from a few months to over 10 years (Traxinger et al., 2013). This wide range of prognoses has greatly impacted the ability to accurately predict disease outcomes. Meanwhile, the considerable diversity in clinical manifestations has posed significant therapeutic challenges for this disorder, with riluzole standing as the sole universally approved clinical treatment and providing a modest survival advantage of 2–3 months (Bensimon et al., 1994).

Even though the primary involvement of motor pathways in ALS has been unequivocally established by post-mortem examinations of the brain, the presence of abnormalities in extra-motor function has introduced an additional layer of complexity to the heterogeneity of the disease. Notably, cognitive and behavioral alterations are reported to occur in 35 to 50% of ALS patients early in the disease progression (Crockford et al., 2018; Beeldman et al., 2020; Bersano et al., 2020; Pender et al., 2020). These changes frequently manifest as impairments in language and executive function. Approximately 15% of ALS cases reach the diagnostic threshold for a frontotemporal dementia (FTD) phenotype (Ringholz et al., 2005; Pender et al., 2020). Moreover, increasing evidence have confirmed psychiatric abnormalities in ALS, including apathy, irritability, depression, anxiety, loss of empathy, sleep disturbances, neglect of personal hygiene, alterations in eating habits, etc. (Beeldman et al., 2020). The presence of extra-motor features, cognitive and behavioral changes in particular, revealed the extensive network of dysfunction in ALS and provide support for the notion that ALS is a multisystem syndrome instead of a pure motor disorder, affecting various domains of the central nervous system beyond classical motor areas. This definition extension has prompted the utilization of advanced neuroimaging techniques to investigate the disease. Structural MRI has proven valuable in detecting both gray matter and white matter alterations associated with ALS, offering potential diagnostic markers (Chiò et al., 2014; Menke et al., 2017). Notably, atrophy of the primary motor cortex, evidenced by thinning of precentral gyrus has been consistently observed in ALS patients (van der Graaff et al., 2011; Agosta et al., 2012; Schuster et al., 2013). In addition, atrophy of the orbitofrontal, temporal, posterior cingulate, parietal, temporal operculum, and cerebellar were also observed in ALS patients in several neuroimaging studies, indicating the possibility of brain MRI as the biomarkers representing distinct disease-specific phenotypes, disease progression, and cognitive profiles (Consonni et al., 2018; Dieckmann et al., 2022; Tan et al., 2022). However, despite the suggestive related patterns of focal cortical atrophy in ALS patients, it remains challenging to reliably interpret the causal relationships between the alterations of the brain structures and ALS development, given the presence of various confounding factors or reverse causation.

Mendelian randomization (MR) is a methodological approach in genetic epidemiology that utilizes genetic variants as instrumental variables (IVs) to investigate causal relationships between modifiable risk factors and disease outcomes (Davey Smith and Hemani, 2014). This method leverages the principles of Mendelian inheritance, that is, the genetic variants are randomly assigned at conception and remain fixed throughout an individual’s life. With these genetic variants as proxies for modifiable risk factors, MR aims to overcome limitations of traditional observational studies, such as confounding and reverse causation (Smith and Ebrahim, 2003; Sleiman and Grant, 2010; Haycock et al., 2016). This approach provides a framework to assess the potential causal effects of exposures on outcomes, offering valuable insights into disease etiology and informing public health interventions. Bayesian colocalization analysis is a statistical method used to assess whether two or more traits or phenotypes share a common genetic basis by calculating the posterior probability of colocalization (Giambartolomei et al., 2014). This approach provides insights into the genetic architecture underlying complex traits and helps identify shared genetic factors contributing to multiple phenotypes.

In this study, we conducted a bidirectional two-sample MR and colocalization analysis using publicly available summary-level genome-wide association study (GWAS) statistics to evaluate the forward and reverse associations between brain structure characteristics and ALS. Our findings provide novel evidence for the concept that the brain morphometry alterations precede ALS onset and these brain structural MRI data have potential to serve as the biomarker for precise medicine-oriented clinical trials.

## 2 Materials and methods

### 2.1 Study design

**Figure 1:**
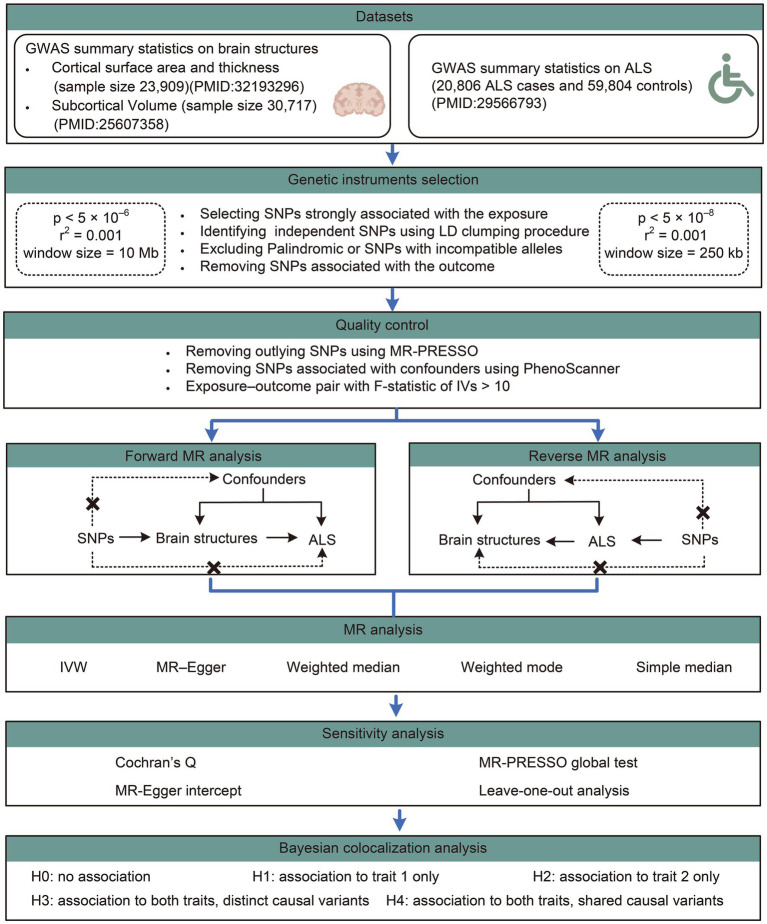
Workflow of the causal inference between brain structures and ALS. ALS, amyotrophic lateral sclerosis; GWAS, genome-wide association study; SNP, single nucleotide polymorphisms.

The study overview was shown in Figure 1. A bidirectional two-sample MR analysis was conducted to investigate the causal associations of cortical structure phenotypes, including cortical surface area (SA), cortical thickness (TH), and subcortical volumes, with ALS. The forward MR analysis was based on the following three assumptions: (i) SNPs used as IVs are strongly associated with the brain structures; (ii) IVs are not associated with any potential confounders that could potentially influence ALS, including type 2 diabetes, lipid profiles (total cholesterol, low-density lipoprotein cholesterol, high-density lipoprotein cholesterol, and triglycerides), adiposity (BMI), physical exercise, smoking, and alcohol consumption; and (iii) IVs are independent of ALS and only affect ALS through modification of the brain structures. The reverse MR analysis was based on the following three assumptions: (i) SNPs used as IVs are strongly associated with ALS; (ii) IVs are not associated with any potential confounders that could potentially influence the brain structures, including neurological and psychiatric disorders, fluid intelligence, obesity, hypoxemia, and other potential confounders (Mulugeta et al., 2021; Song et al., 2021); and (iii) IVs are associated with brain structures only through ALS but not other pathways. Included summary data of the original GWAS used in this study are publicly available, so that no ethical approval from an institutional review board or participant informed consent are required.

### 2.2 Data sources

The GWAS summary data for human cerebral structure traits were obtained from GWAS studies of magnetic resonance imaging (MRI)-derived brain morphometry conducted by the Enhancing NeuroImaging Genetics through Meta-Analysis (ENIGMA) Consortium.^1^ The cortical SA and TH were measured in 51,665 individuals from 60 cohorts across the globe, and data derived from 23,909 participants of European ancestry from 49 cohorts were used in this study (Grasby et al., 2020). A total of 34 brain regions with known functional specializations were defined by Desikan-Killiany atlas, and the regional boundaries are determined by gyral anatomy labeled from the depths of the sulci (Desikan et al., 2006). The SA and TH for the 34 brain regions were averaged across the left and right hemispheres (TH was calculated in mm, and SA in mm^2^). To discern genetic effects specific to each region, the primary GWAS of regional measures incorporated the global measure of SA or TH as a covariate. Notably, data comprising global weighted estimates represented the relative SA and TH measures of specific regions across the entire brain, while those without global weighted estimates represented the SA and TH measures of specific regions, regardless of the global measures of SA and TH. Therefore, TH and SA of the whole cortex, as well as TH and SA for 34 brain cortical regions with or without the weighted estimates of the entire brain yield 138 distinct phenotypes. For the subcortical volumes, seven brain regions were measured in 30,717 individuals from 50 cohorts, including thalamus, nucleus accumbens, putamen, caudate, amygdala, hippocampus, and pallidum, which were all corrected for the intracranial volume (ICV) (Hibar et al., 2015). Volumetric measures were represented as the mean estimates of both hemispheres for the seven structures (calculated in cm^3^).

The GWAS summary data of ALS were obtained from a meta-analysis of GWAS involving 20,806 cases and 59,804 controls (Nicolas et al., 2018). All enrolled ALS patients were of European ancestry, had onset of symptoms after age 18 years, and were diagnosed with probable or definite ALS according to the EI Escorial criteria by a neurologist specializing in ALS. Age and gender were included as covariates to address confounding effects in the primary GWAS analysis.

### 2.3 Genetic instruments selection

In the MR analysis for causal estimation of brain structures on ALS, the threshold of genome-wide significance was set at *p* < 5 × 10^−6^; and linkage disequilibrium (LD) clumping procedure was performed to identify the independent SNPs (R squared threshold = 0.001, window size = 10 Mb) using European 1000G as a reference panel, which was restricted to bi-allelic SNPs with minor allele frequency > 0.01. In the reverse analysis, the genome-wide significant SNPs were identified with the threshold set at *p* < 5 × 10^−8^, LD R squared threshold = 0.001 and window size = 250 kb.

The orientation of alleles in the exposure and outcome datasets were ensured to be consistent by examining allele frequency information. Palindromic SNPs (e.g., those with A/T or G/C alleles) or SNPs with incompatible alleles were excluded from the analysis. SNPs significantly associated with outcomes (*p* < 0.05) were removed. Furthermore, a comprehensive search was conducted in PhenoScanner to retrieve previously established SNPs associated with key factors pertaining to brain structures or ALS.^2^ SNPs linked to the forementioned confounders were excluded from further analysis. The remaining SNPs were then utilized in the MR analysis.

In addition, F -statistics were calculated to evaluate the power of IVs using the following formula as previously described,

$$F=R^{2}\left(N-1-k\right)\left(1-R^{2}\right)k$$

where, R^2^ represents variance of IVs on exposure, N represents the sample size of GWAS for exposure, and k represents the number of IVs. SNPs with F-statistics <10 were removed to avoid weak instrument bias.

### 2.4 Mendelian randomization analyses

TwoSampleMR package (version 0.5.6) for R software (version 4.3.0) was used for the MR analysis in this study. The inverse-variance weighted (IVW) method was utilized as the primary MR approach to investigate causal associations between exposures and outcomes in this study. This method assumes all included SNPs as valid IVs and calculates a weighted average of Wald ratio estimates, providing the most precise results. Four alternative MR methods including MR–Egger, weighted median, weighted mode, and simple median method were further conducted as supplementary methods to calculate estimates for comparison with the IVW estimates. Effect estimates were quantified using β values for continuous outcomes (i.e., cortical structure traits) and were transformed into odds ratio (ORs) for dichotomous outcomes (i.e., ALS status). The false discovery rate (FDR) based on the Benjamini–Hochberg method was applied to adjust for multiple testing. MR Results with the *P*_FDR_ < 0.1 were considered significant; whereas results with *p* < 0.05 but *P*_FDR_ > 0.1 were considered as nominally significant.

To assess the robustness of the primary analyses, a series of sensitivity analysis was conducted. Heterogeneity among different IVs was evaluated using Cochran’s Q statistic (Higgins et al., 2003), where *p* < 0.05 implied significant heterogeneity in the SNP effect estimates. MR-Egger intercept was used to indicate the presence of directional pleiotropy and a potentially invalid IVW estimate when the y-intercept of the regression line significantly different from zero (*p* < 0.05). The I^2^_GX_ statistic was computed to assess heterogeneity among variant-specific causal estimates. An I^2^_GX_ value below 0.9 suggests a higher likelihood of bias toward the null hypothesis in Egger’s method, attributable to the violation of the ‘NO Measurement Error’ (NOME) assumption (Bowden et al., 2016). Assuming 50% of the instruments were valid, MR-Pleiotropy RESidual Sum and Outlier (MR-PRESSO) was used to test for and remove potential “outliers.” The global test value of *p* assessed whether there was any overall horizontal pleiotropy among all IVs. Outlier SNPs identified in the MR-PRESSO analysis were eliminated and MR analysis was recalculated to correct for horizontal pleiotropy. Leave-one-out analysis was performed to identify a single outlying SNP driving the pooled IVW estimates.

### 2.5 Bayesian colocalization analyses

Bayesian colocalization analyses were performed to evaluate the likelihood of two traits sharing the same causal variant using the ‘coloc’ package.^3^ Bayesian colocalization provides the posterior probability for five hypotheses regarding the presence of a single variant shared between the two traits. In this study, we assigned prior probabilities for the SNP being associated exclusively with trait one (p1) as 1 × 10^−4^, the SNP being associated exclusively with trait two (p2) as 1 × 10^−4^, and the SNP being associated with both traits (p12) as 1 × 10^−5^. We assessed the posterior probability of hypothesis four (PPH4), which suggests that both the brain structures and ALS are associated with the region through shared variants. PPH4 values ≥0.75 was defined as a strong indication of colocalization, and 0.5 < PPH4 < 0.75 defined as a moderate indication of colocalization.

## 3 Results

### 3.1 IV selection

For quality control, the IVs associated with potential confounders that interfere with the pathway between brain structures and ALS were removed. Outlier SNPs identified in the MR-PRESSO analysis were also eliminated for further MR analysis. Supplementary material 1 provides a summary of the SNPs used as genetic instruments for both forward and reverse MR analysis. The strength of the instruments was good with all the calculated F-statistics >10.

### 3.2 Forward MR analyses for the causal effects of brain structures on ALS

**Figure 2:**
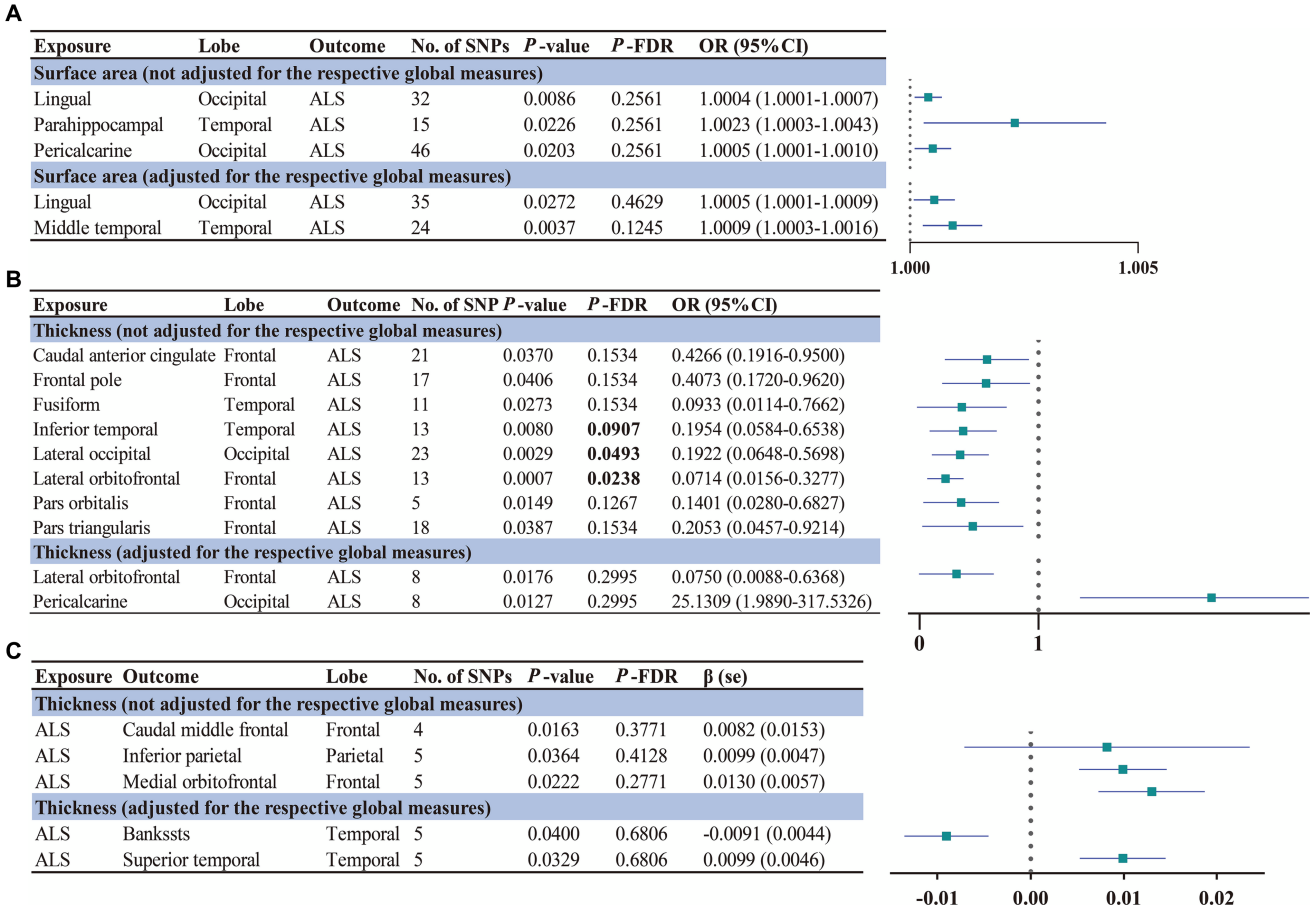
Significant and nominally significant MR estimates for the causal relationship between brain structures and ALS using IVW method. Significant *p*-values after FDR correction (*P*_FDR_ < 0.1) were marked in bold. **(A)** Forward MR analysis for the causal relationship between brain structures SA and ALS risk. **(B)** Forward MR analysis for the causal relationship between brain structures TH and ALS risk. **(C)** Reverse MR analysis for the causal relationship between ALS and brain structures TH. SNP, single nucleotide polymorphism; OR, odds ratio; 95% CI, 95% confidence interval; se, standard error. SA, surface area; TH, thickness; ALS, amyotrophic lateral sclerosis.

**Figure 3:**
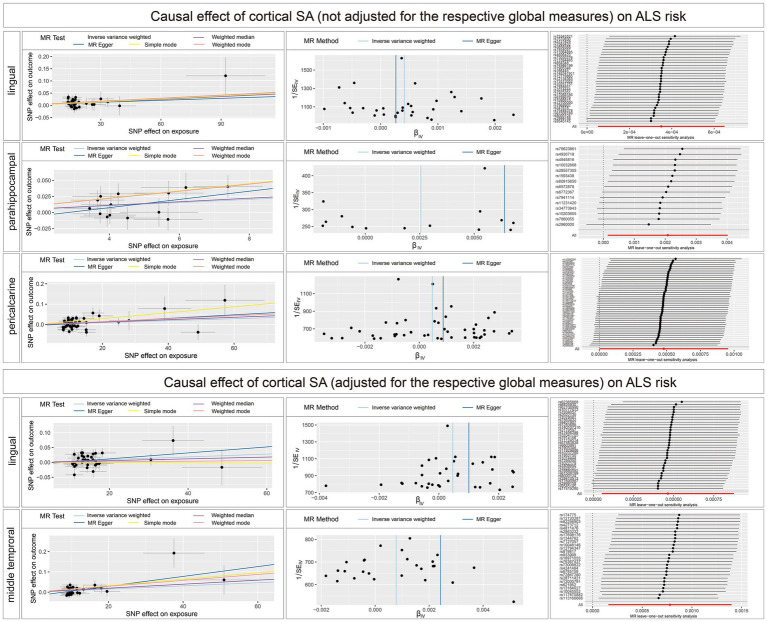
Scatterplots, funnel plots, and leave-one-out sensitivity analysis of the causal effect of brain structures SA on ALS risk. SA, surface area; TH, thickness.

The main findings of the forward and reverse MR analysis are shown in Figure 2. The full results of the causal estimates for all brain regions on ALS are shown in Supplementary material 2; Supplementary Tables S1–S5. Concerning the causal effect of cortical SA on ALS, IVW method showed that increases in the SA of three cortical regions without the weighted estimates of the entire brain were nominally associated with higher risk of ALS (Figure 3), including lingual (OR = 1.0004; 95% CI = 1.0001–1.0007; *p* = 0.0086), parahippocampal (OR = 1.0023; 95% CI = 1.0003–1.0043; *p* = 0.0226), and pericalcarine (OR = 1.0005; 95% CI = 1.0001–1.0010; *p* = 0.0203). After adjustment for the respective global measures, increases in the SA of lingual (OR = 1.0005; 95% CI = 1.0001–1.0009; *p* = 0.0272) and middle temporal (OR = 1.0009; 95% CI =1.0003–1.0016; *p* = 0.0037) were nominally associated with higher risk of ALS (Figure 3).

**Figure 4:**
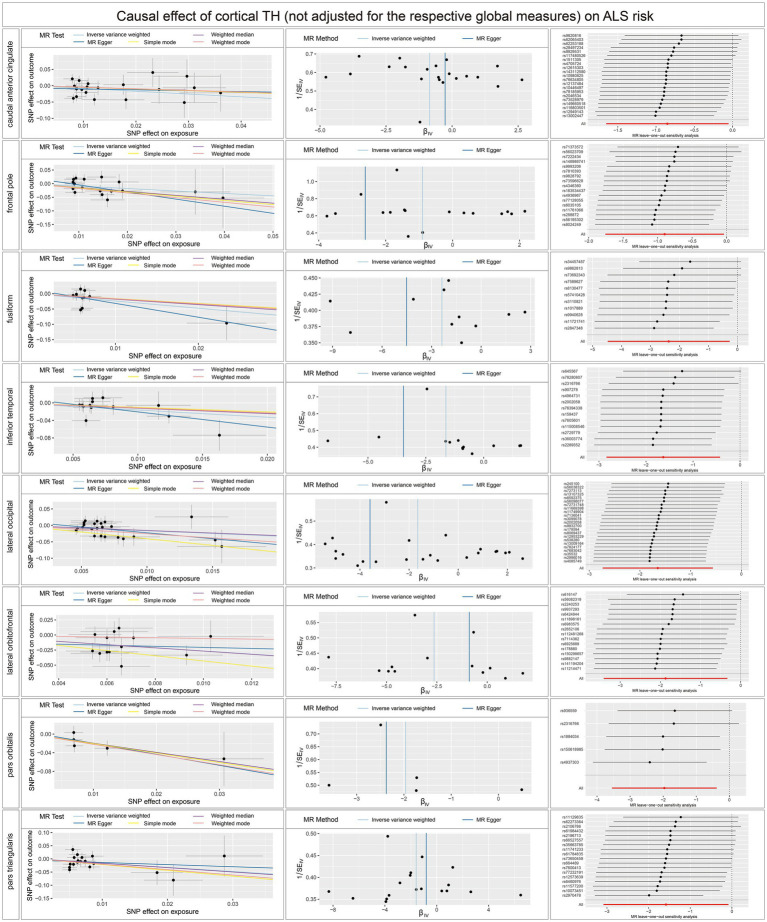
Scatterplots, funnel plots, and leave-one-out sensitivity analysis of the causal effect of brain structures TH (not adjusted for the respective global measures) on ALS risk. SA, surface area; TH, thickness; ALS, amyotrophic lateral sclerosis.

**Figure 5:**
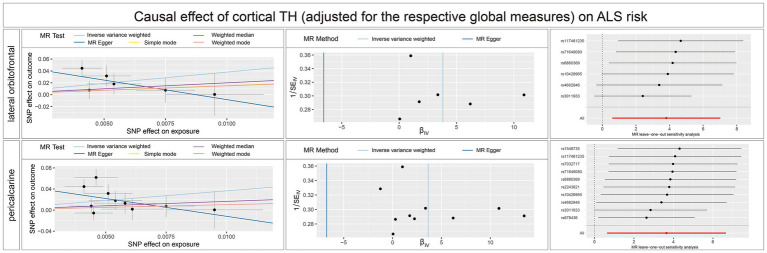
Scatterplots, funnel plots, and leave-one-out sensitivity analysis of the causal effect of brain structures TH (adjusted for the respective global measures) on ALS risk. SA, surface area; TH, thickness; ALS, amyotrophic lateral sclerosis.

Concerning the causal effect of cortical average TH on ALS, increases in cortical average TH (not adjusted for the respective global measures) of inferior temporal (OR = 0.1954; 95% CI = 0.0584–0.6538; *p* = 0.008; *P*_FDR_ = 0.0907), lateral occipital (OR = 0.1922; 95% CI = 0.0648–0.5698; *p* = 0.0029; *P*_FDR_ = 0.0493), and lateral orbitofrontal (OR = 0.0714; 95% CI = 0.0156–0.3277; *p* = 0.0007; *P*_FDR_ = 0.0238) were significantly associated with lower risk of ALS after FDR correction (*P*_FDR_ < 0.1). In addition, we also found nominally significant associations between the increased cortical TH of the caudal anterior cingulate (OR = 0.4266; 95% CI = 0.1916–0.9500; *p* = 0.037), frontal pole (OR = 0.4073; 95% CI = 0.1720–0.9620; *p* = 0.0406), fusiform (OR = 0.0933; 95% CI = 0.0114–0.7662; *p* = 0.0273), pars orbitalis (OR = 0.1401; 95% CI = 0.0280–0.6827; *p* = 0.0149), pars triangularis (OR = 0.2053; 95% CI = 0.0457–0.9214; *p* = 0.0387) and lower risk of ALS (Figure 4). After adjustment for the respective global measures, decreases in the TH of lateral orbitofrontal (OR = 0.0750; 95% CI = 0.0088–0.6368, *p* = 0.0176) and increases in the TH of pericalcarine (OR = 25.1309; 95% CI = 1.9890–317.5326, *p* = 0.0127) were nominally associated with higher risks of ALS (Figure 5). No significant causal associations were found between the seven subcortical volumes (thalamus, nucleus accumbens, putamen, caudate, amygdala, hippocampus, and pallidum) and ALS.

In the sensitivity analysis (Supplementary material 3; Supplementary Tables S1–S5) (Supplementary material 4), no heterogeneity was detected using the Cochran Q test. With the removal of potential outliers identified in the MR-PRESSO global test, no evidence of pleiotropic effects was detected across SNPs in the causal estimates (*p* > 0.05). In addition, the intercepts derived from MR-Egger analysis indicated a minimal likelihood of horizontal pleiotropy. The leave-one-out analysis did not detect any influential single SNPs that could introduce bias to the combined effect estimates.

### 3.3 Reverse MR analyses for the effects of ALS on brain structures

**Figure 6:**
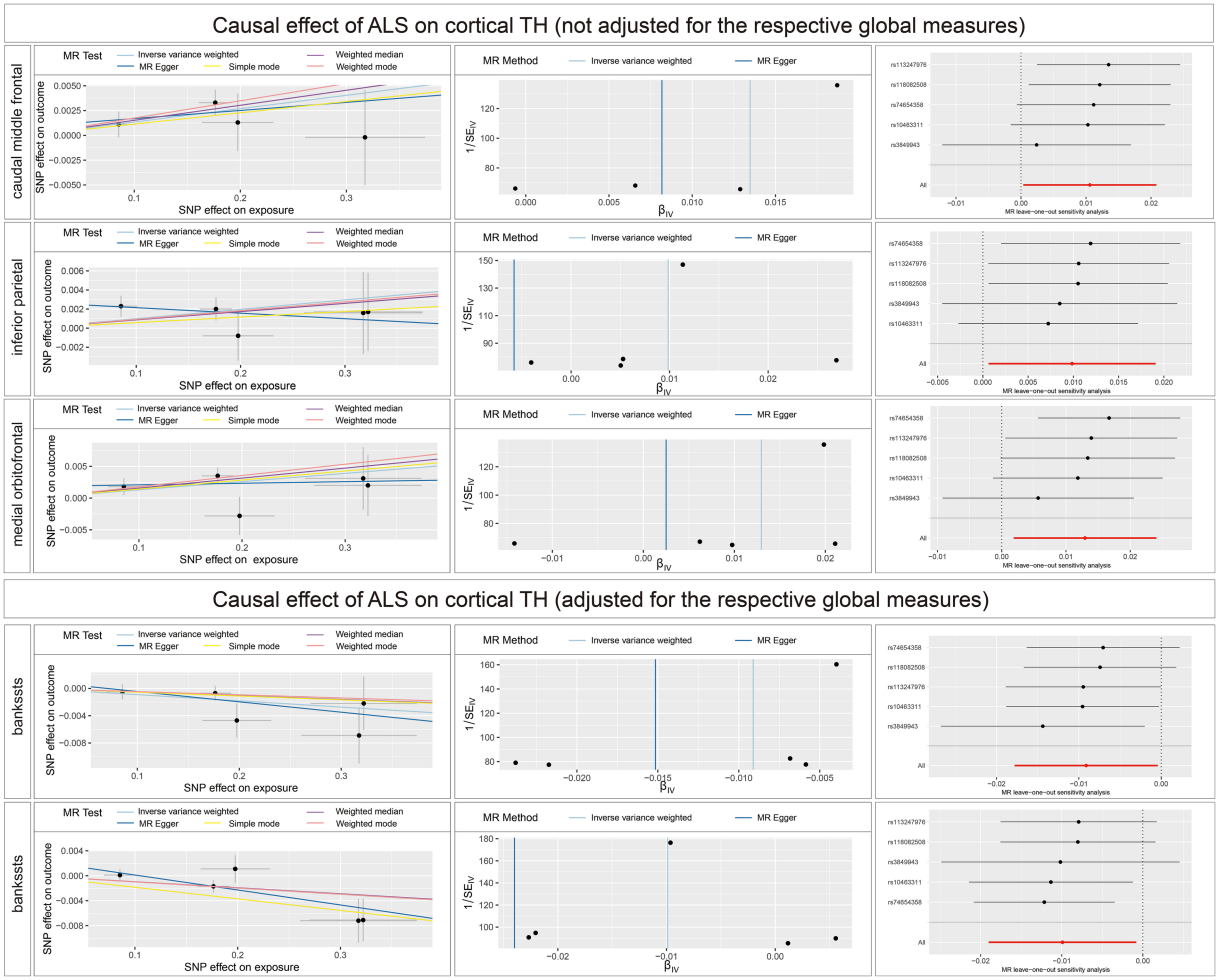
Scatterplots, funnel plots, and leave-one-out sensitivity analysis of the causal effect of ALS on brain structures. SA, surface area; TH, thickness; ALS, amyotrophic lateral sclerosis.

The full results of the causal estimate for the vulnerability to ALS on all brain regions are shown in Supplementary material 2; Supplementary Tables S6–S10. The reverse MR analyses revealed no causal effects of ALS on cortical SA with or without global weighted estimates. As for the cortical TH (Figure 6), the results indicated that genetically predicted ALS was nominally associated with increased cortical TH of the caudal middle frontal (*β* = 0.0082; se = 0.0153; *p* = 0.0163), inferior parietal (*β* = 0.0099; se = 0.0047; *p* = 0.0364), and medial orbitofrontal (*β* = 0.0130; se = 0.0057; *p* = 0.0222) without the weighted estimates of the entire brain. After controlling for the whole brain thickness, genetically predicted ALS was nominally associated with the decreased TH of the bankssts (*β* = −0.0091; se = 0.0044; *p* = 0.0400) and increased TH of superior temporal (*β* = 0.0099; se = 0.0046; *p* = 0.0329). No significant causal associations were found between the genetically predicted ALS and seven subcortical volumes.

Sensitivity analysis (Supplementary material 3; Supplementary Tables S6–S10) (Supplementary material 4) revealed no heterogeneity among the IVs utilized in the reverse causal estimates. No indication of directional or horizontal pleiotropy across SNPs was found in MR-PRESSO global test and MR-Egger test. Additionally, no outlying genetic variants exerted a substantial impact on the estimates in the leave-one-out analysis.

### 3.4 Bayesian colocalization analyses

**Figure 7:**
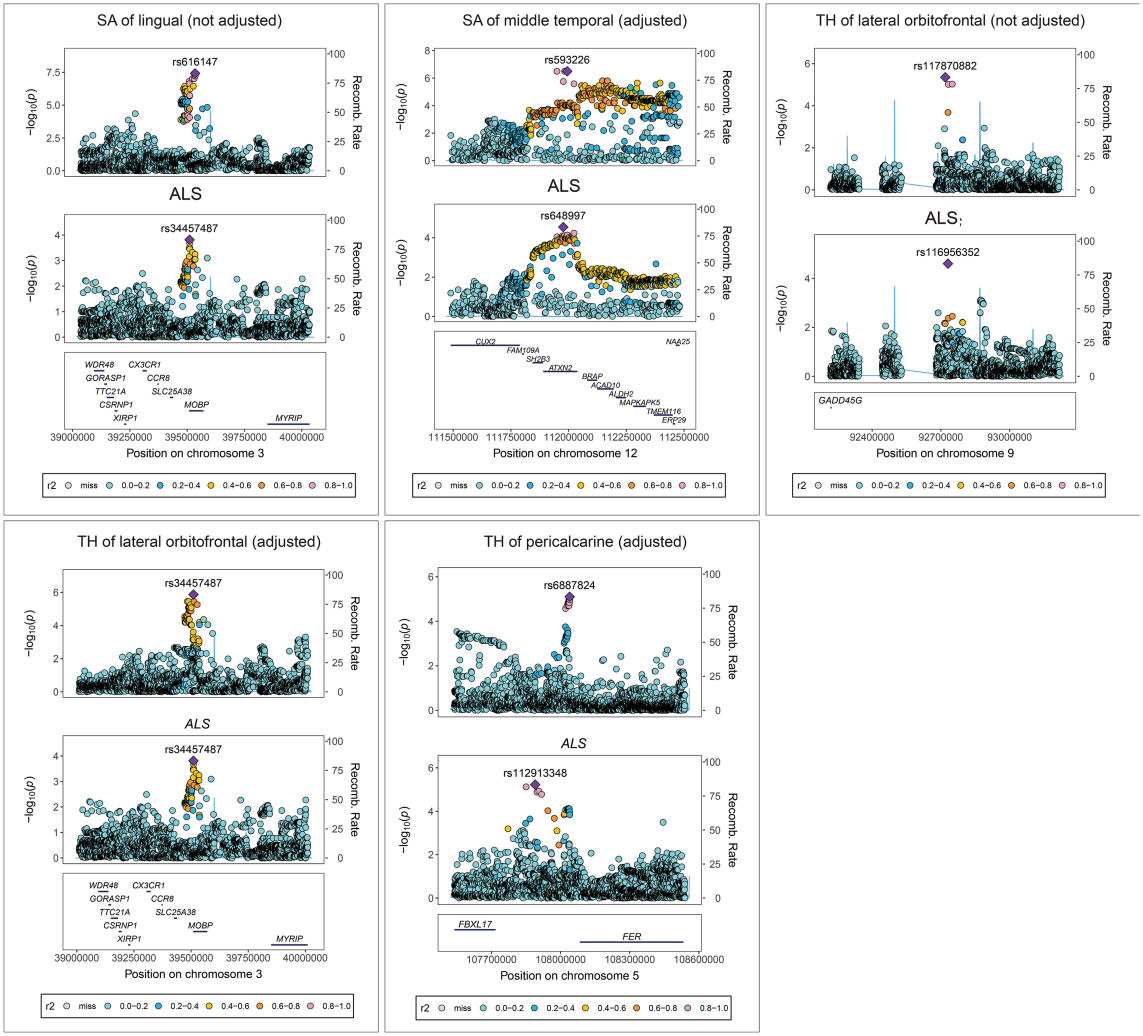
Colocalization analysis results for the association between brain structures and ALS. SA, surface area; TH, thickness; ALS, amyotrophic lateral sclerosis.

Colocalization analysis for the associations between brain structures and ALS with strong or medium indication of colocalization are shown in Figure 7. In detail, TH of pericalcarine (adjusted) and ALS association had a 78.94% PPH4 of sharing a causal variant within the gene region (± 500 kb) of rs6887824. There was also evidence of an association between the SA (not adjusted) of lingual and ALS within the gene region of rs616147 (PPH4 = 63.32%), as well as the SA of middle temporal (adjusted) and ALS within the gene region of rs629404 (PPH4 = 72.63%). In addition, the TH of lateral orbitofrontal (both adjusted and not adjusted) shared the same causal variant with ALS within the region of rs117870882 (PPH4 = 51.53%) and rs34457487 (PPH4 = 57.97%) for colocalization.

## 4 Discussion

Observational studies have reported cognitive deficits in up to 50% of ALS patients, who exhibit various clinical manifestations that span from evident FTD to cognitive impairment falling below the diagnostic criteria for FTD (Crockford et al., 2018; Beeldman et al., 2020; Pender et al., 2020). Anatomical and functional alterations in the cortical and subcortical regions of ALS also have been evidenced using multiple neuroimaging techniques (Consonni et al., 2018; Dadar et al., 2020; Tan et al., 2022). However, prior studies have shown limitations with insufficient statistical robustness due to small sample sizes, diverse interpretations of results, and confounding factors including sociocultural demographic characteristics, psychiatric disorders, aging, obesity, and pre-existing cerebrovascular events. In this study, we utilized MR approach to comprehensively investigate the causal relationships between 145 distinct brain phenotypes and ALS using the large-scale GWAS data that could provide reliable evidence for the associations.

In the forward MR analysis, we identified 15 brain structure phenotypes with a causal influence on ALS. Notably, the increase in the thickness of the seven brain regions in the frontal and temporal lobes (i.e., caudal anterior cingulate, frontal pole, fusiform, inferior temporal, lateral orbitofrontal, pars orbitalis, pars triangularis) was suggestively associated with a decreased risk of ALS, which showed a similar atrophy pattern of ALS-FTD patients with degeneration of frontal and/or temporal lobes as a prominent feature associated with various microscopic changes (Antonioni et al., 2023). It is crucial to acknowledge that both cross-sectional and longitudinal analyses consistently identify the frontal and temporal brain regions as the most probable sources for initiating trans-neural disease progression in ALS (Bhattarai et al., 2022), which further supported the robustness of our main findings.

The anterior cingulate cortex (ACC) engages in prospection, empathy, threat coping, emotional regulation, and self-assessment. The ACC harbors a distinct neuronal structure known as the von Economo neuron, which has been implicated in the pathophysiology of various neuropsychiatric disorders. Previous studies showed that ALS patients with cognitive impairment or FTD exhibited anterior cingulate hypometabolism compared to those with normal cognition (Renard et al., 2011; Cistaro et al., 2014; Canosa et al., 2016). In addition, the pathology of anterior cingulate is demonstrated to be evident in behavioral variant FTD (bvFTD) and associated with an increased risk of death in bvFTD (Antonioni et al., 2023; Harper et al., 2023). Pathological changes in this region have also been identified in autism (Uppal et al., 2014) and schizophrenia (Brüne et al., 2010). Therefore, the negative association of ACC thickness with ALS risk identified in our study suggested its involvement in the neurodegenerative processes specific to ALS cognitive impairment.

The frontal pole locates in the prefrontal region and consists of the most anterior parts of the superior, middle, and inferior frontal gyri. It is an essential region for higher cognitive functions and exhibits strong connections with numerous other brain regions (Snelleksz et al., 2022). The frontal pole is involved in integrating inputs for a common goal, coordinating multitasking, and shifting attention between current and future tasks (Snelleksz et al., 2022). A strong correlation between the quantified parameters representing anomalous decision-making features of ALS patients and decreased degree centrality in the anterior cingulate gyrus and frontal pole was confirmed using resting-state network analysis (Imai et al., 2020). Dysfunction in the frontal pole was also reported to contribute to the pathology of schizophrenia, Alzheimer’s disease, etc. (da Silva Filho et al., 2017; Snelleksz et al., 2022). The orbitofrontal cortex (OFC) is also a prefrontal cortex region that is involved in the cognitive process of decision-making. A volume loss of the orbitofrontal gyri was observed in the ALS patients in multiple studies (Finegan et al., 2021; Temp et al., 2022). Consonni et al. (2019) found that the thinning of the bilateral orbitofrontal cortex was associated with severe apathetic symptoms in ALS using imaging analyses, which was consistent with Tsujimoto’s findings (Tsujimoto et al., 2011). In addition, patients with Parkinson’s disease were demonstrated to exhibit variations in decision-making performance that could be linked to the lateral orbitofrontal cortex (Kobayakawa et al., 2017). Our study indicated that the increases in the thickness of frontal pole and lateral orbitofrontal (even after global adjustment) were suggestively associated with a decreased risk of ALS. Therefore, it can be inferred that the atrophy of the frontal pole and lateral OFC may precede cognitive impairment, potentially contributing to the risk of ALS. Further investigations are needed to explore their functional changes associated with ALS-specific choice behavior.

The pars orbitalis and pars triangularis are situated within the most rostral portion of the frontal lobe, constitute two of the three subdivisions of the inferior frontal gyrus and encompass a diverse range of social, cognitive, and language processing functions. Multiple results of cross-sectional analyses demonstrated a significant atrophy as well as lower baseline activity of inferior frontal gyrus (specifically the pars orbitalis and pars triangularis) in ALS patients compared to controls, which could serve as an early indicator of cognitive decline, particularly executive dysfunction (McMackin et al., 2021; Bhattarai et al., 2022). The identification of negative causal associations of the pars orbitalis and pars triangularis with ALS risk supported their potential involvement in seeding the spread of pathology in ALS.

We also found a causal association between decreased thickness of fusiform and inferior temporal and increased risk of ALS in the forward MR analysis. The fusiform gyrus is situated in the ventral temporal cortex and represents the most prominent macro-anatomical entity within this region, primarily encompassing structures implicated in higher-order visual processing such as face perception, object recognition, and reading (Weiner and Zilles, 2016). The inferior temporal gyrus is located in the anterior region of the temporal lobe and plays a crucial role in processing visual stimuli and memory and recall processes for object identification (Gross, 2008). Consistent with our findings, a notable dissimilarity in cortical thickness within the fusiform gyrus was observed between ALS patients and the control group in a cross-sectional analysis (Schuster et al., 2014). In addition, a correlation was found between the rate of clinical progression and the atrophy observed in the temporal lobes in classical ALS, specifically in the left fusiform gyrus and the right superior temporal sulcus (Giambartolomei et al., 2014). Furthermore, an accurate prediction of atrophy progression within the ALS group over time was achieved by initiating the network diffusion process from the inferior temporal gyrus at the 6-month mark (Walhout et al., 2015). Longitudinal analysis also demonstrated a significant decrease in cortical thickness over time both in the fusiform and inferior temporal (Walhout et al., 2015; Bhattarai et al., 2022). Therefore, in line with previous studies reporting dysfunctional visual processing in ALS patients (Zaino et al., 2023), our findings indicated that the thinning of the fusiform and inferior temporal might be linked to the neurodegenerative pathology in ALS.

Unexpectedly, we found that increased thickness (with global weighted estimates) and SA of the pericalcarine might increase the risk of ALS. There is a paucity of studies examining the role of pericalcarine alterations in the pathogenesis of ALS. The presence of reduced cortical volumes in the pericalcarine regions of patients with early clinically isolated optic neuritis was found to be associated with the subsequent development of multiple sclerosis (Jenkins et al., 2011). Moreover, though atrophy in the pericalcarine cortex were observed in Alzheimer’s disease, a causal relationship between genetically predicted Alzheimer’s disease and increased volume of the pericalcarine was demonstrated using MR analysis that might be contributed to space-occupying effects of amyloid plaques or neurogenesis (Wu et al., 2021). The role of pericalcarine in the course of ALS needs further investigation.

Furthermore, the increase in the SA of middle temporal, lingual, and parahippocampal were suggestively associated with a higher risk of ALS. A significant correlation was observed between the occurrence of spherical or crescent-shaped inclusions in the parahippocampal gyrus and the presence of dementia in ALS cases (Kawashima et al., 2001). It was postulated that the presence of the inclusions might contribute to the cognitive decline in ALS development. On the other hand, higher amplitude of low-frequency fluctuation values that represent brain activity levels were found in the right parahippocampal gyrus in sporadic ALS patients (Zhu et al., 2015). These values were demonstrated to be positively associated with ALS progression rate, and cognitive and executive impairment degrees (Zhu et al., 2015). Therefore, these findings offer evidence of parahippocampal involvement in the risk and progression of ALS, although further evidence is required to strengthen this association. Regarding the negative impact of SA alterations in the middle temporal and lingual regions on ALS, the colocalization analysis supported the MR results. Previous GWAS identified an association between the MOBP (myelin-associated oligodendrocyte basic protein) genetic locus at 3p22.1 (particularly the rs616147), and neurodegenerative diseases such as multiple sclerosis, Alzheimer’s disease, FTD, and progressive supranuclear palsy (Höglinger et al., 2011; Nasrabady et al., 2018). Oligodendrocytes and myelination processes play a crucial role in these diseases, and the MOBP gene may be involved in the pathogenesis of ALS based on changes in myelin composition and pathological findings in ALS subjects. The rs629404 is located within ATXN2, intermediate-length expansions of which is a relatively common cause of heritable ALS (Van Damme et al., 2011). However, the results need to be interpreted with caution due to the relatively small OR values.

In the reverse MR analysis, our findings showed that genetically predicted ALS was causally associated with increased thickness of the caudal middle frontal, inferior parietal, medial orbitofrontal and superior temporal, as well as decreased thickness of bankssts. Intriguingly, though we have found that lateral orbitofrontal atrophy could lead to a higher ALS risk, the inverse MR analysis indicated that genetically predicted ALS could increase the thickness of medial orbitofrontal. This could be explained by the functional heterogeneity within the OFC supported by recent research findings. It’s reported that the medial and lateral regions of the OFC distinctly encode various elements of cognitive maps pertaining to task-related spatial information (Wilson et al., 2014; Schuck et al., 2016). In the context of rodent studies, the lateral OFC encodes the agent’s initial spatial positions within the task map, thereby dictating the set of feasible actions associated with said position (i.e., initial state) (Bradfield and Hart, 2020). Conversely, the medial OFC encodes the agent’s anticipated future spatial positions within the task map, thereby influencing the selection of actions required to attain said position (i.e., the terminal state) (Bradfield and Hart, 2020). These encoding processes exhibit a degree of independence as well as interdependence, and are facilitated by analogous yet non-identical neural circuitry (Bradfield and Hart, 2020). Therefore, we postulated that the atrophy of lateral OFC associated with inferring initial states within tasks may contribute to the ALS risk, whereas the neurodegenerative process in ALS patients may lead to the compensatively activation of neurons in medial OFC associated with terminal states in cognitive map. Moreover, the morphological modifications occurring in distinct areas of the caudal middle frontal, inferior parietal, superior temporal, and bankssts regions could potentially correlate with a multitude of intricate molecular mechanisms underlying the progressive neurodegeneration observed in ALS. These mechanisms encompass aberrant aggregation of TDP-43, inflammatory responses and astroglial activation, synaptic dysfunction and tissue integrity impairment (Illán-Gala et al., 2020; Liu et al., 2023), etc. More longitudinal studies are warranted to elucidate the extent of cortical alterations in individuals diagnosed with ALS.

## 5 Conclusion

Overall, our study revealed significant associations between structural changes in specific functional regions of human brain and susceptibility to ALS. This study highlights distinct patterns of cortical atrophy in ALS and demonstrated that brain structural MRI data could serve as a valid biomarker for the study of extramotor cortical neurodegeneration in the ALS-FTD clinical spectrum. Identified shared genetic loci warranted further investigations into the biological functions of these brain regions and the underlying molecular mechanism of the causal associations.

## 6 Limitations

This study is an exploratory investigation, aiming to evaluate the forward and reverse associations between brain structure characteristics and ALS and offer novel insights into the notion of a continuum between ALS and FTD. Consequently, we implemented an appropriate and sufficiently rigorous, though not the most stringent, quality control approach in conducting the MR estimates to mitigate the risk of potential false negatives. The MR estimates for the TH of pericalcarine (adjusted) in the forward MR analysis and the TH of inferior parietal (not adjusted) in the reverse MR analysis lack consistency across the five methods, so the inferred causality needs to be interpreted cautiously. Future studies are warranted to comprehensively validate these associations, elucidate the underlying mechanisms, and provide robust evidence regarding the potential utility of the brain structural MRI data serving as a valid biomarker for the ALS-FTD clinical spectrum.

## Data availability statement

The original contributions presented in the study are included in the article/Supplementary material, further inquiries can be directed to the corresponding author.

## Ethics statement

The studies involving humans were approved by each cohort enrolled in the original GWAS studies. The studies were conducted in accordance with the local legislation and institutional requirements. Written informed consent for participation was not required from the participants or the participants' legal guardians/next of kin in accordance with the national legislation and institutional requirements.

## Author contributions

JS: Conceptualization, Formal analysis, Writing – original draft, Writing – review & editing. ZW: Conceptualization, Formal analysis, Writing – original draft. MY: Investigation, Writing – review & editing. SX: Formal analysis, Writing – review & editing. XZ: Investigation, Writing – review & editing. DT: Formal analysis, Writing – review & editing. YL: Supervision, Writing – review & editing. YY: Supervision, Writing – review & editing.

## Footnotes

^1^ https://enigma.ini.usc.edu/

^2^ www.phenoscanner.medschl.cam.ac.uk

^3^ https://github.com/chr1swallace/coloc
